# A rare case of paraganglioma of the cystic duct

**DOI:** 10.1016/j.ijscr.2018.09.041

**Published:** 2018-10-01

**Authors:** Raha AlMarzooqi, Loay AlJaberi, Steven Rosenblatt, Thomas Plesec, Eren Berber

**Affiliations:** a Department of General Surgery, Cleveland Clinic, 9500 Euclid Ave., Cleveland, Ohio, 44195, United States; b Al-Quds University School of Medicine, East Jerusalem, Palestine; c Department of Pathology, Cleveland Clinic, 9500 Euclid Ave., Cleveland, Ohio, 44195, United States; d Department of Endocrine Surgery, Cleveland Clinic, 9500 Euclid Ave., Cleveland, Ohio, 44195, United States

**Keywords:** Case report, Cystic duct, Neuroendocrine tumor, Paraganglioma

## Abstract

**Introduction:**

Biliary system paragangliomas are rare neuroendocrine tumors of embryonic neural crest origin. The majority is asymptomatic and incidentally found due to gallbladder functional disorders. Herein, we present a non-functional, 2.25 mm focus in the cystic duct, which to our knowledge, is the first reported paraganglioma of the cystic duct.

**Presentation of case:**

The patient presented to the Emergency Department complaining of a sudden-onset, right upper abdominal and epigastric pain. Ultrasound and Computed Tomography were both consistent with signs of early cholecystitis. Laparoscopic cholecystectomy was performed without major complications. In addition to cholelithiasis and chronic cholecystitis, pathological examination reported a neuroendocrine proliferation in the cystic duct measuring 2.25 mm favoring paraganglioma. Incidentally, the patient is unique in that they were also found to have an adrenal nodule and a normocalcemic primary hyperparathyroidism that raised suspicion for an underlying endocrinopathy. Nevertheless, genetic testing was negative.

**Discussion:**

Extensive literature review demonstrates only nine cases of gallbladder paraganglioma, and three cases of hepatic ducts paraganglioma, but no cases of paraganglioma occurring at the cystic duct. Although a gene mutation and syndrome was not identified in the patient, the fact that an adrenal nodule and normocalcemic primary hyperparathyroidism were present, suggests that a complete hormonal workup should be obtained in these patients.

**Conclusion:**

It is important to realize that biliary system paragangliomas, although rare, may occur. As they have an association with multiple endocrine neoplasia syndrome, a thorough endocrine investigation should be made.

## Highlights

• This is the first reported case of cystic paraganglioma in the literature.
• Paragangliomas in the differential of gallbladder or cystic/hepatic duct lesions.
• Importance of thorough investigation, due to association with endocrinopathies.

## 1 Introduction

Paragangliomas are rare neuroendocrine tumors of neural crest origin that arise from chromaffin cells. Paragangliomas can be potentially found anywhere along the paravertebral axis from their predominant location at the base of the skull and neck to the pelvis [1]. Pheochromocytoma and carotid body tumor are the two most common types of paragangliomas, which occur in the adrenal medulla and at the bifurcation of the carotid artery, respectively; however, remaining paragangliomas are usually retroperitoneal in origin, and found in the sympathetic or parasympathetic ganglia [1,2]. Most paragangliomas are asymptomatic and present as a painless mass [1]. While all can potentially secrete hormones such as catecholamines due to their origin from chromaffin cells, only a small percentage of cases are clinically significant and evoke systemic symptoms [1]. Biliary system paragangliomas are predominantly seen in females in the fifth to sixth decade of life [3]. These tumors are typically discovered incidentally during gallbladder or unrelated surgery, or secondary to complications such as obstructive jaundice, right upper quadrant pain, and gastrointestinal bleeding [3]. Herein, we present a non-functional, 2.25 mm focus in the cystic duct, which to our knowledge, is the first reported paraganglioma of the cystic duct. This work has been reported in line with the SCARE criteria [4].

## 2 Presentation of case

**Fig. 1:**
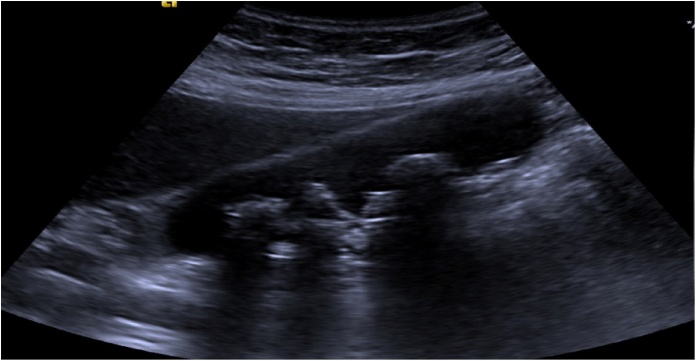
Ultrasound mass showed signs of early cholecystitis.

**Fig. 2:**
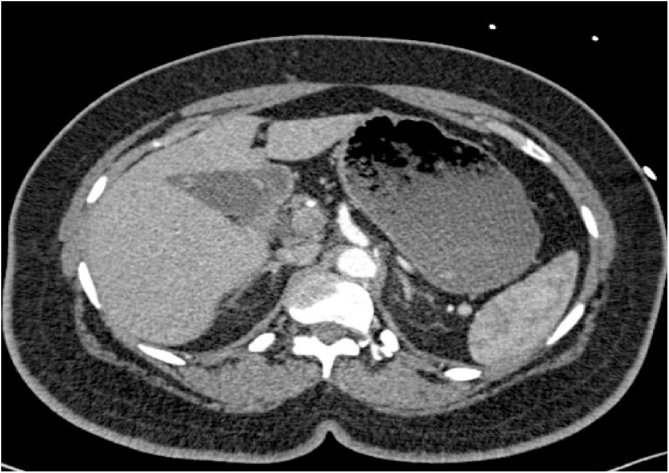
CT scan at the time of original presentation of the patient.

A patient with a past medical history of atrial fibrillation, hypertension, and hyperlipidemia and no past surgical history walked into the Emergency Department of an academic institute complaining of a sudden-onset, sharp, right upper abdominal and epigastric pain radiating to the back. Family history was negative and social history did not include any tobacco, alcohol, or drug misuse. On physical examination, the patient was tender in the right upper quadrant. On imaging, a right upper quadrant ultrasound showed signs of early cholecystitis (Fig. 1) and computed tomography of the abdomen showed gallstones and distended liver bile ducts, distended gallbladder with wall thickening, edema, and a mild surrounding inflammation consistent with the ultrasound findings (Fig. 2). Anti-coagulation was held and the patient underwent an uncomplicated laparoscopic cholecystectomy two days after presentation by the general surgery team. Intra-operatively, an inflamed appearing gallbladder was noted. The patient was discharged on the second postoperative day and recovered uneventfully.

**Fig. 3:**
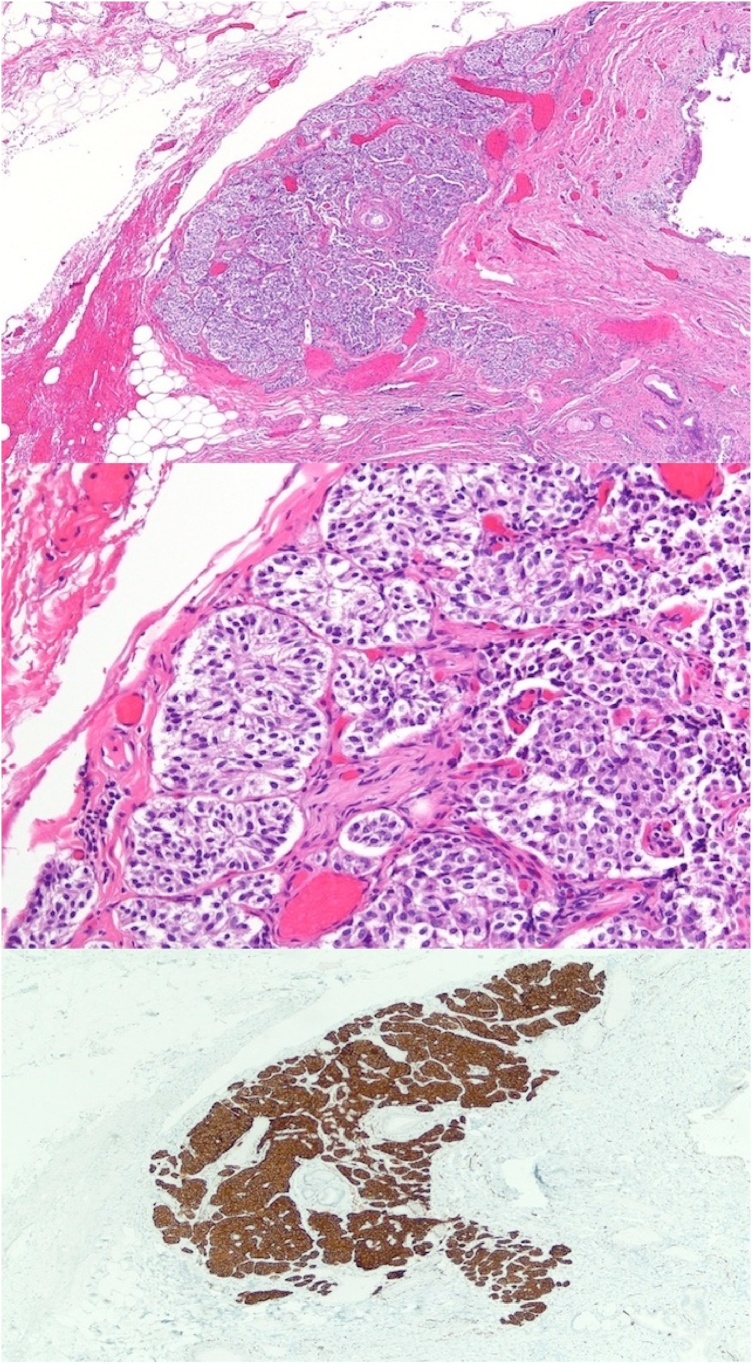
(a) Low power photomicrograph of the lesion in the center of the image. Part of the cystic duct lumen is seen at the right edge of the micrograph (hematoxylin and eosin stain, original magnification ×40). (b) Medium power photomicrograph demonstrating the characteristic nested growth pattern of small, monomorphic cells that contain pale-to-eosinophilic foamy cytoplasm (hematoxylin and eosin stain, original magnification ×200). (c) Low power photomicrograph revealing diffuse and strong immunopositivity for synaptophysin (original magnification ×40), supporting the morphologic impression of neuroendocrine differentiation.

On pathology, routine hematoxylin and eosin-stained sections revealed a relatively well-circumscribed 2.25 mm lesion adjacent to the cystic duct. The lesion was composed of nests of monomorphic cells containing pale-to-eosinophilic foamy cytoplasm. The nuclei were round, with granular chromatin and lacked significant mitotic activity. No necrosis was identified. On immunohistochemical studies, the specimen showed diffuse positivity for synaptophysin and focal positivity for chromogranin, supporting neuroendocrine differentiation. SOX-10 highlighted a few cells at the periphery of the nests, consistent with sustentacular cells. In all, the morphologic and immunophenotypic findings were most consistent with an incidental paraganglioma (Fig. 3a–c).

Based on this pathology report, in the follow-up appointment a few weeks later, the patient was referred to an endocrine surgeon. The patient had blood and urine work-up including a plasma catecholamine panel showing epinephrine levels of 20 pg/mL (10–200), norepinephrine levels of 492 pg/mL (80–520), and dopamine of <20 pg/mL (0–20), all within normal levels. The patient also had a 24 h urine metanephrine panel showing metanephrine levels of 169 ug/24 h (52–341), and normetanephrine levels of 333 ug/24 h (88–444), all within normal levels. Chromogranin A level was 181 ng/mL, which was high, normal less than 95 (ranging from 0 to 95).

**Fig. 4:**
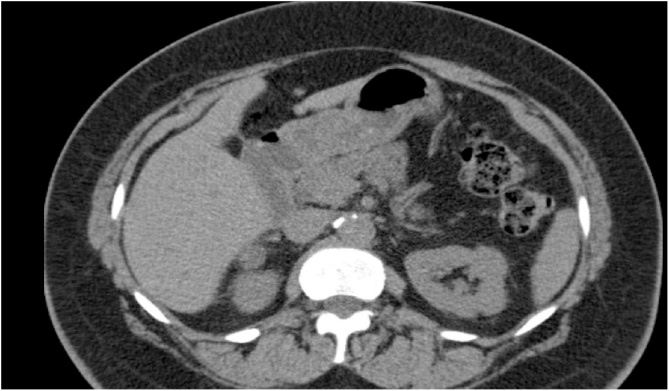
Non-contrast CT showing a 1.5 cm nodule involving the adrenal gland with a Hounsfield unit density of 14. On MRI, this was interpreted as a benign adenoma.

As a part of the patient’s investigations, computed tomography scan of the chest, abdomen, and pelvis was ordered. The scan demonstrated a 1.5 cm right adrenal mass with a Hounsfield unit of 14 (Fig. 4). The left adrenal gland was normal. MRI revealed a benign adenoma. The patient subsequently underwent blood and urine work-up including plasma ACTH and cortisol, as well as 24 h urine cortisol which were all normal at 8 pg/mL (<47), 10.3 ug/dL (AM = 5.3–22.5, PM = 3.4–16.8), and 23 ug/d (<45) respectively. In addition, the patient underwent a plasma renin and 24 h urine aldosterone work-up, which were also normal at 10.4 pg/ml (Upright: 3.6–81.6 pg/mL, Supine: 2.5–45.1 pg/mL) and 13 ug/24 h (3–25) respectively, with a 24 h urine sodium of 171 mmol/24 h (40–220) and potassium of 52 mmol/24 h (30–99). Finally, her work-up included a 24 h urine catecholamine panel showing epinephrine levels of 5 ug/d (1–7), norepinephrine levels of 37 ug/d (16–71), and dopamine levels of 140 ug/d (77–324).

Work-up for primary hyperparathyroidism was also initiated in the next few months. Labs showed calcium of 9.5 mg/dL (8.5–10.2) with a PTH of 73 pg/mL (15–65), and a 25 hydroxyvitamin D level of 32.9 ng/mL (31–80).

Finally, a hereditary paraganglioma-pheochromocytoma panel and evidence genes through Invitae was performed. Genetic testing did not reveal any mutations of EGLN1, FH, KIF1B, MAX, MEN1, NF1, RET, SDHA, SDHAF2, SDHB, SDHC, SDHD, TMEM127, or VHL genes.

## 3 Discussion

A literature review demonstrates nine cases of gallbladder paraganglioma [3,[5], [6], [7], [8], [9], [10]], with the first reported case in 1972 by Miller at al [5], and three cases of hepatic paraganglioma [[11], [12], [13]], with the first reported case in 1980 by Sarma et al [11]. All reported paraganglioma cases were non-functional. Further features of these paragangliomas are summarized in the tables below (Table 1, Table 2).

**Table 1:** Paragangliomas of the Gallbladder.

	Clinical Presentation	Imaging Findings	Location	Size
Miller et al. [5]	Recurrent hematemesis	Duodenal ulcer	Unknown	3 cm
Wolff [6]	Cholelithiasis	Unknown	Subserosal	Unknown
Wolff [6]	Cholelithiasis	Unknown	Subserosal	Unknown
Wolff [6]	Cholelithiasis	Unknown	Subserosal	Unknown
Hirano [7]	RUQ pain	Mass at the neck of the gallbladder	Submucosal	1.3 cm
Cho et al. [8]	RUQ pain	Mass at the fundus of the gallbladder	Unknown	2.5 cm
Mehra et al. [3]	Asymptomatic	None	Subserosal	1.5 cm
Rodriguez-Merchan et al. [9]	RUQ pain	Intra and extra-hepatic biliary dilation	Subserosal	1 cm
Ece et al. [10]	RUQ pain	Mass at the neck of the gallbladder	Serosa and muscularis propria	1.8 cm

**Table 2:** Paragangliomas of the Hepatic Duct.

	Clinical Presentation	Imaging Findings	Location	Size
Sarma et al. [11]	Obstructive jaundice	Mass at the hepatic duct	Hepatic duct	Unknown
Hitanant et al. [12]	Obstructive jaundice	Extrahepatic biliary dilation	Hepatic duct	5 × 2 × 1.8 cm
Carceres et al. [13]	RUQ pain	Unknown	Hepatic duct	Unknown

However, extensive literature review revealed no cases of paraganglioma occurring at the cystic duct. In this case report, the patient presented with classical signs and symptoms of acute cholecystitis. Pre-operatively, the patient had no symptoms indicating a paraganglioma and the focus of the tumor was too small to be seen on imaging. It was only on pathological review of the specimen that a neuroendocrine proliferation measuring 2.25 mm in size at the cystic duct was noticed, which further stains favored a paraganglioma. Incidentally, the patient is unique in that they were also found to have an adrenal nodule, and a mild normocalcemic primary hyperparathyroidism that raises suspicion for an underlying endocrinopathy, despite a negative gene panel.

Although a gene mutation and syndrome was not identified in this patient, the fact that an adrenal nodule and normocalcemic primary hyperparathyroidism was present, suggests that a complete hormonal workup should be obtained in patients that present with paragangliomas.

## 4 Conclusion

It is important to realize that biliary system paragangliomas, although rare, may occur. Paragangliomas should be considered in the rare differential diagnosis of gallbladder, cystic duct, and hepatic duct lesions. Paragangliomas of the biliary system have been reported to be benign, malignant, and to have an association with multiple neuroendocrine neoplasia (MEN) syndrome [1]. Therefore, a thorough endocrine investigation to rule out this association should be made.

## Conflicts of interest

The authors have no conflicts to declare.

## Funding

There was no source of funding.

## Ethical approval

This is case report is exempt for ethical approval in our institute.

## Consent

Written informed consent was obtained from the patient for publication of this case report and accompanying images. A copy of the written consent is available for review by the Editor-in-chief of this journal on request.

## Author contribution

Raha AlMarzooqi, Loay AlJaberi: Wrote and revised manuscript.

Thomas Plesec, Steven Rosenblatt, Eren Berber: Reviewed manuscript.

## Registration of research studies

Not applicable.

## Guarantor

Eren Berber.

## Provenance and peer review

Not commissioned, externally peer-reviewed.
